# The association between Annexin A2 and epithelial cell adhesion molecule in breast cancer cells

**DOI:** 10.1002/cnr2.1498

**Published:** 2021-07-09

**Authors:** Saad Misfer Al‐Qahtani, Salah Eldin Gadalla, Min Guo, Christer Ericsson, Daniel Hägerstrand, Monica Nistér

**Affiliations:** ^1^ Department of Oncology‐Pathology Karolinska Institutet Stockholm Sweden; ^2^ Department of Pathology, College of Medicine and Najran University Hospital Najran University Najran Saudi Arabia; ^3^ iCellate Medical AB Stockholm Sweden

**Keywords:** ANXA2, breast cancer, EpCAM, molecular pathology, tPA

## Abstract

**Background:**

The epithelial cell adhesion molecule (EpCAM) is a type I transmembrane and glycosylated protein, which is overexpressed in many neoplasms. However, EpCAM has no known ligand partners and the mechanisms by which it functions are not fully understood.

**Aim:**

This study was performed to discover novel partners of EpCAM, which may provide a better understanding of its functions.

**Methods:**

The membrane fraction of the ERα^+^ noninvasive breast cancer cell line ZR‐75‐1 and MCF‐7 was extracted and followed by co‐immunoprecipitation of EpCAM using C‐10, a mouse monoclonal antibody raised against amino acids 24–93 of the EpCAM molecule. As a negative control, MDA‐MB‐231 and Hs578T were used since they express a negligible amount of EpCAM and are known as EpCAM^−/low^ ERα^−/low^ invasive and tumorigenic breast cancer cell lines.

**Results:**

Annexin A2 (ANXA2) was found to be selectively and differentially co‐immunoprecipitated with EpCAM in the ERα^+^ breast cancer cells MCF‐7 and ZR‐75‐1. ANXA2 is a multifunctional protein and known to act as a co‐receptor for tissue plasminogen activator (tPA) on the surface of endothelial and cancer cells, thereby affecting fibrinolytic activity and neoangiogenesis as well as invasive and metastatic properties. In this study, the association between EpCAM and ANXA2 was found to affect the activity of tPA.

**Conclusion:**

This study concludes that ANXA2 co‐localizes with EpCAM at the plasma membrane, and the co‐localization may have functional implications. Data suggest that EpCAM supports ANXA2 to function as a co‐receptor for the tPA, and that EpCAM has a regulatory function on the expression and subcellular localization of ANXA2.

## 1 INTRODUCTION

The epithelial cell adhesion molecule (EpCAM) is a type I transmembrane and glycosylated protein which is overexpressed in many neoplasms including high grade glial tumors,^1^ breast cancer,^2^ and colorectal cancer.^3^ However, there are complicated roles for EpCAM in the different cancers which may be attributed to the fact that the binding partners of EpCAM and the mechanisms by which it signals in and out of the cell are not fully known.^4^, ^5^ Therefore, EpCAM co‐immunoprecipitation was performed and followed by mass spectrometry and peptide sequencing to search for new potential binding partners of EpCAM. The endoplasmic reticulum aminopeptidase 2 (ERAP2) has been identified by our group as an EpCAM‐associated protein,^6^ and here we continue this effort and present another novel finding that Annexin A2 (ANXA2) is a potential interacting partner of EpCAM in the EpCAM^+^ ERα^+^ breast cancer cells.

Annexins are a family of proteins with more than 22 members that have been isolated from a variety of cells and tissues and are involved in diverse physiological activities. Annexins consist of a variable amino terminal “tail” domain followed by four or eight conserved repeats.^7^ Annexins in general bind to biological membranes and anionic phospholipids in a Ca^2+^ dependent manner through these conserved repeats. ANXA2 belongs to this family of membrane binding proteins, and it controls apical plasma membrane and lumen formation.^8^ The protein may cross‐link plasma membrane phospholipids with actin and the cytoskeleton and be involved in exocytosis, membrane and vesicular trafficking.

ANXA2 was discovered by many investigators independently and given a variety of names (p34, p36, p39, calpactin I heavy chain, protein I, chromobindin‐8, lipocortin II and placental anti‐coagulant protein IV).^9^, ^10^ The protein exists in two major forms in cells, as a heterotetramer and as a 36 kDa monomer. A heterotetramer of ANXA2 contains two light chains of S100A10/p11 and two heavy chains of AnxA2/p36 and localizes to the cell surface.^11^, ^12^

The tetramer exists in the sub‐plasma lemmal cytoskeletal network in different cell types.^13^, ^14^ As a monomer, ANXA2 is found in both the cytosol and nucleus, but predominantly in the cytosol.^15^, ^16^ The function of the ANXA2 monomer in the nucleus was suggested by its purification as part of a primer recognition protein complex that enhances DNA polymerase α activity in vitro.^17^, ^18^, ^19^

Through S100A10, ANXA2 has been reported to function as a co‐receptor for tissue plasminogen activator (tPA) at the cell surface.^20^ In a previous report, it was shown that depletion of ANXA2 in telomerase immortalized microvascular endothelial cells led to the loss of plasminogen binding and plasmin generation similar to when S100A10 was depleted.^21^ Furthermore, analysis of *AnnexinA2*‐null mice showed that tPA‐dependent plasmin generation at the endothelial cell surface is markedly deficient in the absence of ANXA2.^22^ The phenotype of these mice showed that ANXA2 is a regulator of cell surface plasmin generation and that impaired endothelial cell fibrinolytic activity constitutes a barrier to effective neoangiogenesis.

The tPA is one of the proteases that convert the plasminogen to the active plasmin, and plasmin is involved in the degradation of the extracellular matrix.^23^ The degradation of the extracellular matrix is a reported mechanism for progression of cancer, invasion and metastasis.^24^

This study presents a novel finding that ANXA2 is an interacting partner of EpCAM in the EpCAM^+^ ERα^+^ breast cancer cells, and co‐localizes with EpCAM at the plasma membrane of EpCAM^+^ ERα^+^ breast cancer cells. This co‐localization may have a functional background since EpCAM appeared to support ANXA2 to function as a co‐receptor for the tPA, and EpCAM seemed to have a regulatory function on the expression of ANXA2.

## 2 MATERIAL AND METHODS

### 2.1 Cell lines

MCF‐7, ZR‐75‐1 and MC2 are ERα^+/high^ and EpCAM^+/high^ breast cancer cell lines while MDA‐MB‐231 and Hs578T are ERα^−/low^ and EpCAM^−/low^.^25^ MC2 is a line of ERα transfected MDA‐MB‐231 cells. All cell lines were grown in d‐MEM with 10% fetal calf serum, 1% l‐glutamine, 1% sodium pyruvate (all from GIBCO) and 1% penicillin/streptomycin (SIGMA). All of the breast cancer cell lines were provided by Professor Jonas Bergh's laboratory at the Department of oncology‐pathology, Karolinska Institutet, Stockholm, Sweden except MC2 which was provided by Professor V. Craig Jordan, Fox Chase Cancer Center's Division of Medical Science, USA. All the cell lines were used in two previous publications from our group.^6^, ^26^ In those studies we could confirm their expected expression patterns of several breast cancer proteins as well as their phenotypes.

### 2.2 Subcellular fractionation and EpCAM co‐immunoprecipitation followed by gel staining, band picking and mass spectrometry

#### 2.2.1 Subcellular fractionation

MCF‐7, ZR‐75‐1, MDA‐MB‐231 and Hs578T cells were grown on 6‐well plates up to 70%–80% confluence, and each cell line in duplicate. Then the cells were washed three times on ice with cold PBS and scraped off, cells were then spun down for 5 min at 700 G, 4°C. Cell pellets were re‐suspended in homogenization buffer (250 mM sucrose, 10 mM Tris–HCl buffer, pH 7.4). Homogenization was performed by drawing and releasing the suspended cells many times using 22 G needle and 10 cc syringe and the material was then spun down for 15 min at 1500 G, 4°C. The supernatant constitutes cytosolic proteins and membrane proteins, and it was then transferred to Sorval™ and centrifuged 10 600 rcf for 1 h at 4°C. The supernatant represents cytosolic fraction while the pellet is membrane fraction.

#### 2.2.2 Immunoprecipitation

The membrane fraction of the MCF‐7, ZR‐75‐1, MDA‐MB‐231 and Hs578T cells was solubilized in ice‐cold RIPA buffer 1:10 volume (RIPA buffer: 150 mM NaCl, 0.5% sodium deoxycholate, 0.1% SDS, 50 mM Tris, pH 8.0, 1% Non‐Idet P‐40/NP‐40), and the suspension was centrifuged at 16 000 rcf for 15 min at 4°C. To clear any unbound immune complexes, the supernatant was incubated with protein G agarose beads at 4°C for 2 h (Immunoprecipitation starter pack, GE Healthcare), beads were spun down by centrifuging for 3 min at 3000 rcf and the supernatant was incubated with mouse monoclonal anti‐human EpCAM (C‐10, Santa Cruz) at a dilution of 2 μg per 100–500 μg of total protein (in 1 mL of cell lysate) overnight at 4°C. Immune complexes were captured with protein G agarose beads for 2 h at 4°C, eluted with 50 μL 2× SDS sample buffer, shaken at 95°C for 10 min and centrifuged. The resulting supernatant represents EpCAM with possible co‐immunoprecipitated proteins.

#### 2.2.3 SDS‐PAGE

Immunoprecipitated (IP) protein samples were run in 10% SDS‐PAGE, and for all cell lines analyzed the IP lane was compared to the total cell extract (input). Gel was stained overnight by mild shaking in Colloidal Blue Staining Kit from Invitrogen, it was destained by deionized water over 8–12 h, where after differentially expressed bands were identified, gel slices were cut out and sealed in a clean plastic bag and sent for ESI‐MALDI MS–MS and peptide mass fingerprinting (Proteomics Resource Center, Department of Medical Biochemistry and Microbiology, Biomedical Center, Uppsala University, Sweden). Finally, querying NCBI Protein, OMIM, Swissprot and ExPASy identified proteins.

### 2.3 Validation of protein–protein association by reciprocal Co‐IP and Western blotting in ZR‐75‐1 cells

Total cell extracts were prepared from the cell cultures after washing with cold PBS three times, and solubilization in RIPA buffer and co‐immunoprecipitation was performed as described above, except that immunoprecipitates were captured on magnetic beads. The immunoprecipitates were dissolved in 2× SDS. Total protein concentration was measured using the Bio‐Rad RCDC assay and 50 μg total protein per lane was loaded on to 10% SDS PAGE gel and separated. The separated proteins were transferred overnight to nitrocellulose membrane (Schleicher & Schuell Protran BA 83) using wet Western blot transfer. The membrane was washed with TBS‐T (25 mM Tris–HCl, pH 8.0, 125 mM NaCl, 0.1% Tween 20) and then blocked for 1 h with 10% membrane blocking agent in PBS (Amersham). Incubation with the primary antibody (the mouse monoclonal C‐10 against EpCAM alternatively the H‐50 rabbit polyclonal antibody against ANXA2 [both from Santa Cruz]) was overnight at 1:2000 dilution at 4°C. The membrane was washed and subsequently incubated with the appropriate secondary antibody for 1 h at room temperature. After washing with TBS‐T, a mixture of Amersham ECL Western blotting detection reagent 1 and 2 was poured onto the membrane and incubated for 1 min. Luminescence was imaged by Fuji Film LAS‐1000 and images analyzed using LAS‐1000 and Image Gauge software. This experiment was conducted twice and independently.

### 2.4 Subcellular co‐localization of ANXA2 and EpCAM by double immunofluorescence staining and confocal microscopy

MCF‐7, ZR‐75‐1, MC2, MDA‐MB‐231 and Hs578T cells were cultured on coverslips in six‐well plates. Each cell line was seeded in four wells (*n* = 4), incubated as described above and this experiment was performed twice and independently. After reaching 70%–80% confluency, the cell cultures were washed gently twice in PBS and fixed in 96% ethanol for 15 min at room temperature. After washing twice in PBS, cells were permeabilized with 0.5% Triton X‐100 in PBS for 15 min followed by blocking in 10% BSA in PBS for 1 h at 4°C, then incubated overnight in a humid chamber at 4°C with the mouse monoclonal C‐10 against EpCAM and the H‐50 rabbit polyclonal antibody against ANXA2 (both from Santa Cruz). Second day the slips were washed three times in PBS for 40 min and incubated with the corresponding conjugated secondary antibodies, DyLight 594‐conjugated anti‐mouse IgG (red) and DyLight 488‐conjugated anti‐rabbit IgG (green) (Both from Vector Labs) in 1:1000 dilution for 1 h at 4°C and then washed three times with PBS at room temperature. For visualizing the nuclei, a drop of Vectashield mounting media with DAPI (Vector Labs) was added. Then, TCS SP5 confocal microscopy system (Leica) was used to visualize and examine the cells.

### 2.5 Transfection of siRNA constructs

MCF‐7, ZR‐75‐1, MDA‐MB‐231 and Hs578T cells were cultured in six‐well plates. Silencer™ Select Pre‐Designed siRNA (Thermo Scientific) was used to silence both ANXA2 (Catalog#: 4390826, siRNA ID: s1383) and EpCAM (Catalog #: 4392422, siRNA ID: s8370). As a negative control, Silencer™ Select Negative Control (Thermo Scientific, Catalog #4390844) was used. This was in the presence of another control group of cells that was not treated at all, and each group of the controls and siRNA‐treated group was grown in four wells (*n* = 4). The transfection was carried out using Lipofectamine‐2000 reagents (Invitrogen) and OPTI‐MEM serum free media (GIBCO). After 24 h of incubation at 37°C, the transfection was repeated, and the cells were harvested after another 48 h for further studies including assessment of relative gene expression and protein levels as described below.

### 2.6 Relative gene expression: RNA extraction, cDNA synthesis, and qPCR using SYBR Green

#### 2.6.1 RNA extraction and cDNA synthesis

After the transfection, total RNA was extracted using the RNeasy mini kit from Qiagen, and the amount of RNA was assessed by NanoDrop (Thermo Scientific, USA). The cDNA from each sample was then synthesized using 1 μg of RNA added to previously mixed 1 μL of Oligo (dT) 12–18 bp primers (Invitrogen) and 1 μL of dNTP Mix (Invitrogen), incubated for 5 min at 65°C and reaction stopped by incubation on ice for 1 min. After that the mixture was added to a premix that was made of 4 μL of 5× first single strand buffer, 1 μL of RNaseOut Recombinant, 1 μL of 0.1 M DTT, and 1 μL of Superscript III RT (all from Invitrogen). The generated cDNA was diluted and kept at −20°C.

#### 2.6.2 qPCR using SYBR Green

The qPCR was done using the 2XSYBR Green Master Mix Kit (Applied Biosystem), and all the primers were obtained from Invitrogen (Table 1). The relative expression was calculated by the ∆∆Ct method after calculating the average since each sample in each group was assessed in triplicate (*n* = 4). The normalized Ct values (∆Ct) were calculated by subtracting the Ct values of the target genes from that of GAPDH. The ∆∆Ct values were then calculated by subtracting the normalized value for the target gene from that of the control, and the formula 2**^** ^−ΔΔCT^ was used to calculate the relative expression of the target gene in comparison to the control which was given the value 1.

**TABLE 1:** The primers used during the assessment of the relative expression levels

Primer	Sequence (5′–3′)	Amplicon size
F‐ANXA2	GAGCGGGATGCTTTGAACATT	119
R‐ANXA2	TAGGCGAAGGCAATATCCTGT	
F‐EpCAM	AATCGTCAATGCCAGTGTACTT	178
R‐EpCAM	TCTCATCGCAGTCAGGATCATAA	
F‐GAPDH	GGAGCGAGATCCCTCCAAAAT	197
R‐GAPDH	GGCTGTTGTCATACTTCTCATGG	

### 2.7 Western blot

After the transfection of siRNA and control, proteins were extracted using radioimmunoprecipitation assay (RIPA) buffer, and the amount of the protein quantified according to a standard assay protocol (DC protein assay, Bio‐Rad, USA). About 50 μg of each sample prepared with SDS‐loading buffer and proteins were separated on NUPAGETM 4%–12% Bis–Tris protein gels (Invitrogen). Each sample from each group (*n* = 4) were run in duplicates. Then, proteins were transferred to PVDF membranes using Trans‐Blot Turbo transfer system (Bio‐Rad). Membranes were blocked with 5% non‐fat milk in PBS and incubated with the primary antibodies (Table 2) over night. The next day, after washing several times with TBS‐T, membranes were incubated with the appropriate peroxidase‐conjugated secondary antibody (GE Healthcare) for 1 h. Finally, membranes were immersed with the Pierce ECL Western Blotting Substrate (Thermo Scientific), and luminescence was imaged by Fuji Film Las‐1000 and images analyzed using ImageJ.

**TABLE 2:** The antibodies were used during the study of protein levels

Antibody	Source	Clone	Provider
Anti‐EpCAM	Mouse monoclonal	C‐10	Santa Cruz
Anti‐ANXA2	Rabbit polyclonal	H‐50	Santa Cruz
Anti‐GAPDH	Mouse monoclonal		Santa Cruz
HRP‐conjugated anti‐mouse	Sheep	Secondary	GE healthcare
HRP‐conjugated anti‐rabbit	Donkey	Secondary	GE healthcare

### 2.8 tPA chromogenic activity assay

Using tPA activity assay kit (Colorimetric, Human ab108905, Abcam), the amount of unbound tPA was assessed according to the manufacturer's protocol. MCF‐7 and ZR‐75‐1 were treated with siRNA‐cont, siRNA‐EpCAM, siRNA‐ANXA2, or both siRNA‐EpCAM and siRNA‐ANXA2; in presence of control groups that were not treated at all. MDA‐MB‐231 and Hs578T were transfected by either siRNA‐cont or siRNA‐ANXA2, in presence of control groups that were not treated at all as well. After 72 h of siRNA transfection, the cell culture supernatants from each well were collected and centrifuged to remove debris. Using the microplate provided by the manufacturer, 80 μL of the Assay Mix (60 μL diluent, 10 μL plasminogen, 10 μL plasmin substrate) added to 20 μL of each sample (cell culture supernatant) or 20 μL of different concentrations of tPA standard (16, 8, 4, 2, and 0 IU/mL). Each group (*n* = 3) was assessed in triplicates except control groups and standards that were assessed in duplicates. Then, the absorbance at 405 nm at zero minutes was read, and the microplate was incubated in humidified incubator at 37°C. The absorbance at 405 nm was assessed periodically every 30 min for 2 h. In parallel, the cells were used to analyze protein levels, and the extraction of protein was performed directly after removing the supernatants. This experiment was performed twice and independently.

### 2.9 Statistical analyses

All results were generated from at least two independent experiments. Data were analyzed using the Prism Graph Pad Software (CA, USA). All values are presented as mean ± *SD*. Significances were calculated using analysis of variance (ANOVA), followed by multiple comparisons. The values of *p* <.05 were considered significant whereas * indicates *p*‐value <.05, **p‐value <.01 and ***p‐value <.001.

## 3 RESULTS

### 3.1 Co‐immunoprecipitation of ANXA2 with EpCAM in breast cancer cells

**FIGURE 1:**
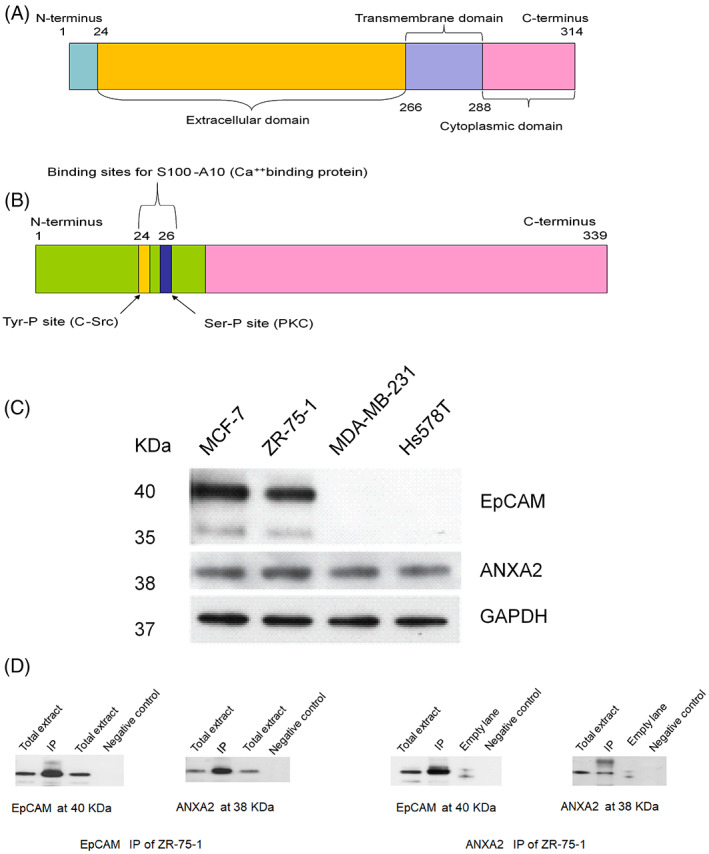
(A) Schematic illustration of human EpCAM protein domain structure. It is formed of 314 amino acids, and there are three domains: extracellular (the largest), transmembrane and cytoplasmic domain. (B) Schematic illustration of human ANXA2 protein domain structure. It is formed of 339 amino acids. The domain for S100A10/p11 binding as well as known sites for regulatory tyrosine phosphorylation by pp60src and serine phosphorylation by PKC are all localized in the N–terminal domain as illustrated. (C) EpCAM and ANXA2 proteins in breast cancer cell lines. Western blot analysis of total cell lysates from breast cancer cells using EpCAM, ANXA2, and GAPDH antibodies. This experiment was repeated twice and independently. (D) and (E) Reciprocal co‐immunoprecipitation of EpCAM and ANXA2 in total cell extracts from ZR‐75‐1 breast cancer cells. EpCAM co‐immunoprecipitation followed by Western blotting using ANXA2 antibodies confirmed an association between the two proteins in the ERα+ breast cancer cell line ZR‐75‐1. Notice that EpCAM appears as a band around 40 kDa while ANXA2 is slightly smaller, with a band at 38 kDa. This study was performed twice and independently, and plain beads without antibodies were added to the extract as a negative control

The gel used to separate the proteins after immunoprecipitation and from which slices were sent for the mass spectrometry is provided as a Figure S1 which was published as part of our coauthor's doctoral thesis.^27^ Additionally, in the mass spectrometry analysis of ZR‐75‐1 cells, several proteins were found co‐immunoprecipitated with EpCAM. Peptide finger printing was done, matched with NCBI protein database, Swissprot and ExPASy. Those proteins showing a statistically significant score are listed in Table 3. ANXA2 was identified with a score of 163/66 in the EpCAM^+^ ZR‐75‐1 cell line (Tables 3 and 4), while it was not picked up by EpCAM co‐immunoprecipitation followed by mass spectrometry analysis in MDA‐MB‐231 cells in which EpCAM is known to be almost absent (not shown). To confirm this finding, we first identified ANXA2 as a 38 kDa protein present at similar levels in ERα^+^ and ERα^−^ breast cancer cells (Figure 1(C)). Then EpCAM co‐immunoprecipitation was repeated, and the precipitates were analyzed by Western blotting using ANXA2 antibodies. The reciprocal co‐immunoprecipitation was performed using ANXA2 antibodies followed by Western blot analysis of EpCAM in the precipitates. This confirmed association between EpCAM and ANXA2 in ZR‐75‐1 cells (Figure 1(D),(E)).

**TABLE 3:** Summary of findings from mass spectrometry and peptide mass fingerprinting

Name of the protein	Symbol	Score	Function
Actin B	ACTB	93/64	Non muscle cytoskeletal actins, highly conserved proteins, involved in cell motility
Keratin 18	CK18	174/64	Intermediate filament that acts as structural cytoskeleton
Keratin 8	CK8	123/64	Intermediate filament that acts as structural cytoskeleton
Keratin 9	CK9	89/64	Intermediate filament that acts as structural cytoskeleton
Keratin 1	CK1	65/64	Intermediate filament that acts as structural cytoskeleton
Keratin 19	CK19	311/64	Intermediate filament that acts as structural cytoskeleton
Heat shock protein 70 9B	HSP70 9b	108/64	Heat related protein that is involved in the folding and unfolding of other proteins
Heat shock protein 90 AA1	HSP90 AA1	112/64	Involved in the folding and unfolding of other proteins
Heat shock protein 70 8 isoform 2	HSP70 8 2	71/64	Involved in the folding and unfolding of other proteins
Myosin (non‐muscle)	Myosin	285/64	Cross links actin (motor function)
Vimentin	VIM	296/64	Type III intermediate filament (IF) protein that is expressed in mesenchymal cells
heterogeneous nuclear ribonucleoprotein A2/B1	hnRNP A2	152/64	RNA binding protein, influences pre‐mRNA processing and other aspects of mRNA metabolism and transport in the nucleus
Fatty acid synthase	FASN	174/66	A multi‐enzyme, catalyzes fatty acid synthesis
Clathrin heavy chain I	CLTC	126/66	A major protein component of coated vesicles, involved in the intracellular trafficking of receptors and endocytosis
ANXA2	ANXA2	163/66	Membrane trafficking, anticoagulation through binding tPA, cell adhesion by binding tenascin C
ERAP2 (LRAP)	ERAP2	73/66	ERAP2 is an aminopeptidase set in the endoplasmic reticulum (ER), a single‐pass type II membrane protein that plays a central role in peptide trimming. Major histocompatibility complex (MHC) class I molecules rely on aminopeptidases such as ERAP2 to trim precursors to antigenic peptides in ER. ERAP2 preferentially hydrolyzes the basic residues Arg and Lys
Valyl‐tRNA synthetase	VARS	81/66	Catalyzes the aminoacylation of tRNA by their cognate amino acids (protein synthesis)
Eukaryotic translation elongation factor 2	EEF2	198/66	GTP‐binding translation elongation factor, involved in protein synthesis
Valosin‐containing protein	VCP	198/66	ATP‐binding proteins involved in vesicle transport and fusion
Enolase 1 (alpha‐enolase)	ENO1	107/66	A glycolytic enzyme
Aldolase A	Aldolase A	94/66	An enzyme that catalyzes a reverse aldol reaction: The substrate, fructose 1,6‐bisphosphate (F‐1,6‐BP) is broken down into glyceraldehyde 3‐phosphate and dihydroxyacetone phosphate (DHAP). This reaction is a part of glycolysis
Lactate dehydrogenase A	LDHA	112/66	An enzyme that catalyzes the conversion of L‐lactate and NAD+ to pyruvate and NADH in the final step of anaerobic glycolysis
Seryl‐tRNA synthetase	SARS	136/66	An enzyme that catalyzes the transfer of L‐serine to tRNA

*Note*: Data are based on EpCAM co‐immunoprecipitates from the membrane fraction of ZR‐75‐1 cells. Information on protein function is from the NCBI‐OMIM database.

**TABLE 4:** Primary data from the mass spectrometry‐based identification of ANXA2 in EpCAM co‐immunoprecipitates

Accession	Mass	Score	Description
1. gi | 4757756	38 808	163	Annexin A2 isoform 2 [Homo sapiens]
2. gi | 56966699	38 866	163	Chain A, Annexin A2: Does It Induce
3. gi | 18645167	38 780	163	Annexin A2 [Homo sapiens]
4. gi | 16306978	38 822	163	Annexin A2 [Homo sapiens]
5. gi | 73909156	40 731	161	Annexin A2 [Homo sapiens]

### 3.2 Co‐localization of ANXA2 and EpCAM in breast cancer cells

**FIGURE 2:**
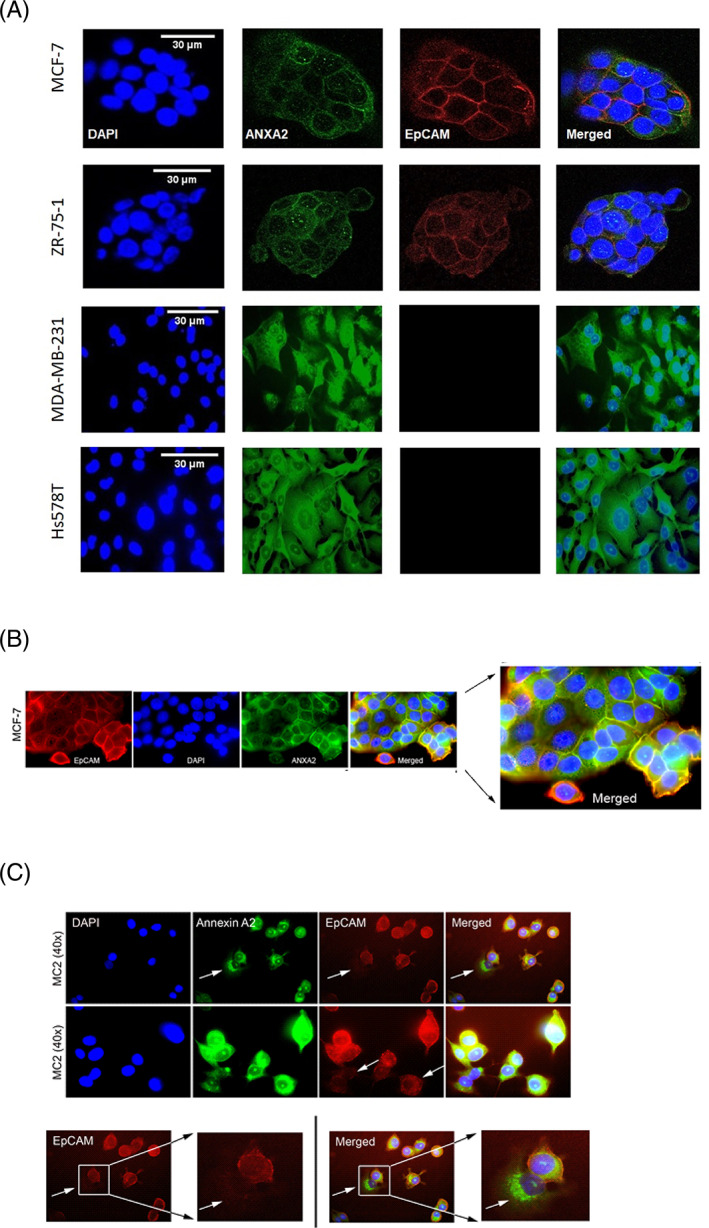
The images are representative of each respective group (*n* = 4), and the experiment was conducted twice and independently. (A) Co‐localization of ANXA2 and EpCAM by double immunofluorescence staining of breast cancer cells. The subcellular localization of EpCAM and ANXA2 is illustrated by double immunofluorescence staining. Note the absence of EpCAM in MDA‐MB‐231 and Hs578T cells and positive immunostaining in MCF‐7 and ZR‐75‐1. In EpCAM positive cells the two proteins co‐localize mainly in a membranous pattern and ANXA2 is additionally present in the cytoplasm and nucleoli of all the cell lines tested. (B) Special view to illustrate co‐localization of EpCAM and ANXA2 in lamellae and membrane ruffles (yellow) at the cell periphery of MCF‐7 cells. (C) Different subcellular localization of ANXA2 in EpCAM+ and EpCAM− MC2 cells. MC2 cells expressing a lesser amount of EpCAM (white arrows) have their ANXA2 mainly localized in the cytoplasm and in these cells the membranous co‐localization in merged pictures is almost lacking. (D) A magnified panel to show MC2 cells that express a lesser amount or nothing of EpCAM (white arrows), compared to the MC2 that expresses EpCAM

Double immunofluorescence staining was performed to determine the subcellular localization of the two proteins. ANXA2 (green) displayed cytoplasmic as well as nucleolar staining patterns in all tested breast cancer cell lines, both in ERα^+^ and ERα^−^ cells (Figure 2(A)). However, ANXA2 in ERα^+^ cells was clearly distributed at the plasma membrane more than in the cytoplasm and the nucleus in which it had a dot‐like appearance; while in ERα^−^ cells ANXA2 appeared diffusely in cytoplasm and nucleus (Figure 2(A)). EpCAM (red) showed membranous localization in MCF‐7, ZR‐75‐1 and a subset of MC2 cells but was not detected in MDA‐MB‐231 and Hs578T cells. ANXA2 and EpCAM co‐localized as a line or dots, mainly at the plasma membrane of the ERα^+^ cells (Figure 2(A)). Also, peripheral lamellae and membrane ruffles displayed ANXA2‐EpCAM co‐localization as illustrated in MCF‐7 (Figure 2(B)), in addition to the most distinct plasma membrane co‐localization in places where cells were closely attached to each other. In MC2 cells, which are originally MDA‐MB‐231 cells engineered to express ERα,^28^ ANXA2‐EpCAM co‐localization was also seen more diffusely in lamellae, at the periphery of cells (Figure 2(C)). In the MC2 cultures where only a subset of cells expressed EpCAM, ANXA2 tended to be localized only in the cytoplasm of EpCAM^−^ cells, while also at the cell periphery of EpCAM^+^ cells (Figure 2(D)).

### 3.3 The mRNA, protein levels and co‐localization of ANXA2 and EpCAM in breast cancer cells after silencing EpCAM and/or ANXA2

**FIGURE 3:**
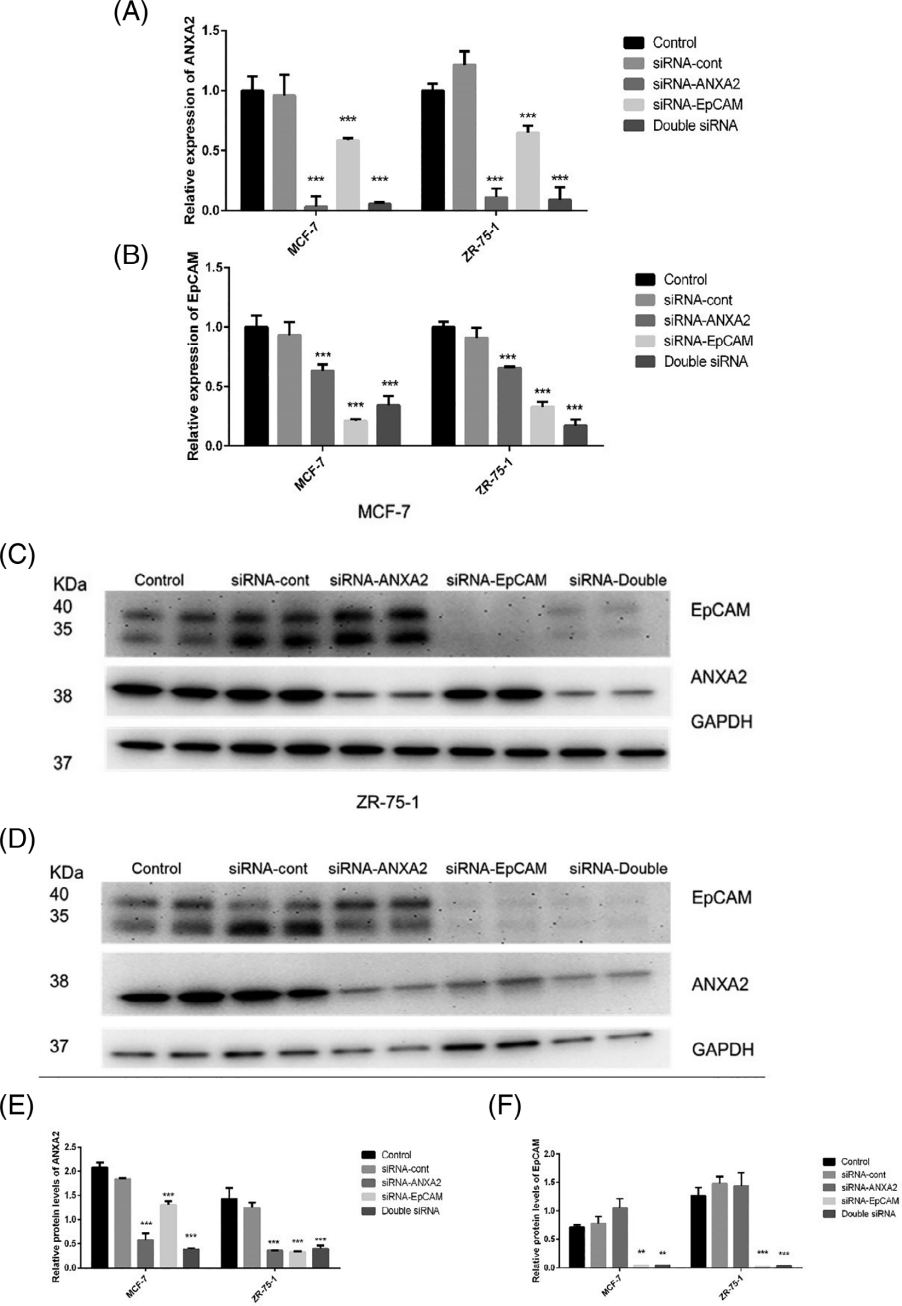
Each group was assessed in triplicates (mRNA levels) or duplicates (protein levels) (n = 4). The values of *p* <.05 were considered significant whereas * indicates *p*‐value <.05, **p‐value <.01 and ***p‐value <.001. (A) The mRNA levels of ANXA2 in MCF‐7 and ZR‐75‐1: The control siRNA did not change the levels, compared to the control groups. However, siRNA‐ANXA2 and/or siRNA‐EpCAM led to a significant reduction in the mRNA levels of ANXA2. (B) The mRNA levels of EpCAM in MCF‐7 and ZR‐75‐1: The control siRNA did not change the levels, compared to the control groups. In contrast, siRNA‐ANXA2 and/or siRNA‐EpCAM led to a significant reduction in the mRNA levels of EpCAM. (C) Immunoblotting shows protein levels of EpCAM, ANXA2, and GAPDH in MCF‐7: control, control‐siRNA, siRNA‐ANXA2 group, siRNA‐EpCAM, and double‐siRNA targeted both ANXA2 and EpCAM. (D) Immunoblotting shows protein levels of EpCAM, ANXA2, and GAPDH in ZR‐75‐1: control, control‐siRNA, siRNA‐ANXA2 group, siRNA‐EpCAM, and double‐siRNA targeted both ANXA2 and EpCAM. (E) The protein levels of ANXA2 normalized to GAPDH in MCF‐7 and ZR‐75‐1: In consistenence with mRNA levels in panel A, the control siRNA did not change the levels, compared to the control groups. However, siRNA‐ANXA2 and/or siRNA‐EpCAM led to a significant reduction in the protein levels of ANXA2. (F) The protein levels of EpCAM normalized to GAPDH in MCF‐7 and ZR‐75‐1: The control siRNA and siRNA ANXA2 did not change the levels of EpCAM, compared to the control group. Meanwhile, siRNA‐EpCAM and the double‐siRNA led to a significant reduction in the protein levels of EpCAM

Using siRNA against ANXA2 (siRNA‐ANXA2) (Figure 3(A)) and EpCAM (siRNA‐EpCAM) (Figure 3(B)) in MCF7 and ZR‐75‐1 resulted in a significant reduction of their respective mRNA levels, compared to the control groups (Figure 3(A),(B)). In the ERα^−/low^ and EpCAM^−/low^ cells, the mRNA levels of ANXA2 were suppressed significantly upon siRNA treatment, in comparison to the control group treated with negative control siRNA (Figure S2(A)). Noteworthy, the significant reduction in the mRNA levels of ANXA2 due to siRNA‐ANXA2 was accompanied by a significant reduction in ANXA2 due to siRNA‐EpCAM (Figure 3(A)). Moreover, the significant suppression of the mRNA levels of EpCAM due to siRNA‐EpCAM treatment was paralleled by significantly suppressed levels of EpCAM due to siRNA‐ANXA2 (Figure 3(B)).

At protein levels, siRNA‐ANXA2 resulted in a significant reduction of ANXA2 in all cells MCF‐7, ZR‐75‐1 (Figure 3(C)–(F)), MDA‐MB‐231 and Hs578T (Figure S2(B),(C)). Also, siRNA‐EpCAM reduced the protein levels of EpCAM significantly in MCF‐7 and ZR‐75‐1 (Figure 3(C)–(F)). Consistent with the observed mRNA levels, the immunoblotting showed that both siRNA‐EpCAM and siRNA‐ANXA2 reduced the protein levels of ANXA2 significantly in MCF7 and ZR‐75‐1 (Figure 3(C)–(E)). However, the observed protein levels of EpCAM were not consistent with its mRNA levels; EpCAM protein levels were not affected by siRNA‐ANXA2 (Figure 3(C),(D),(F)).

**FIGURE 4:**
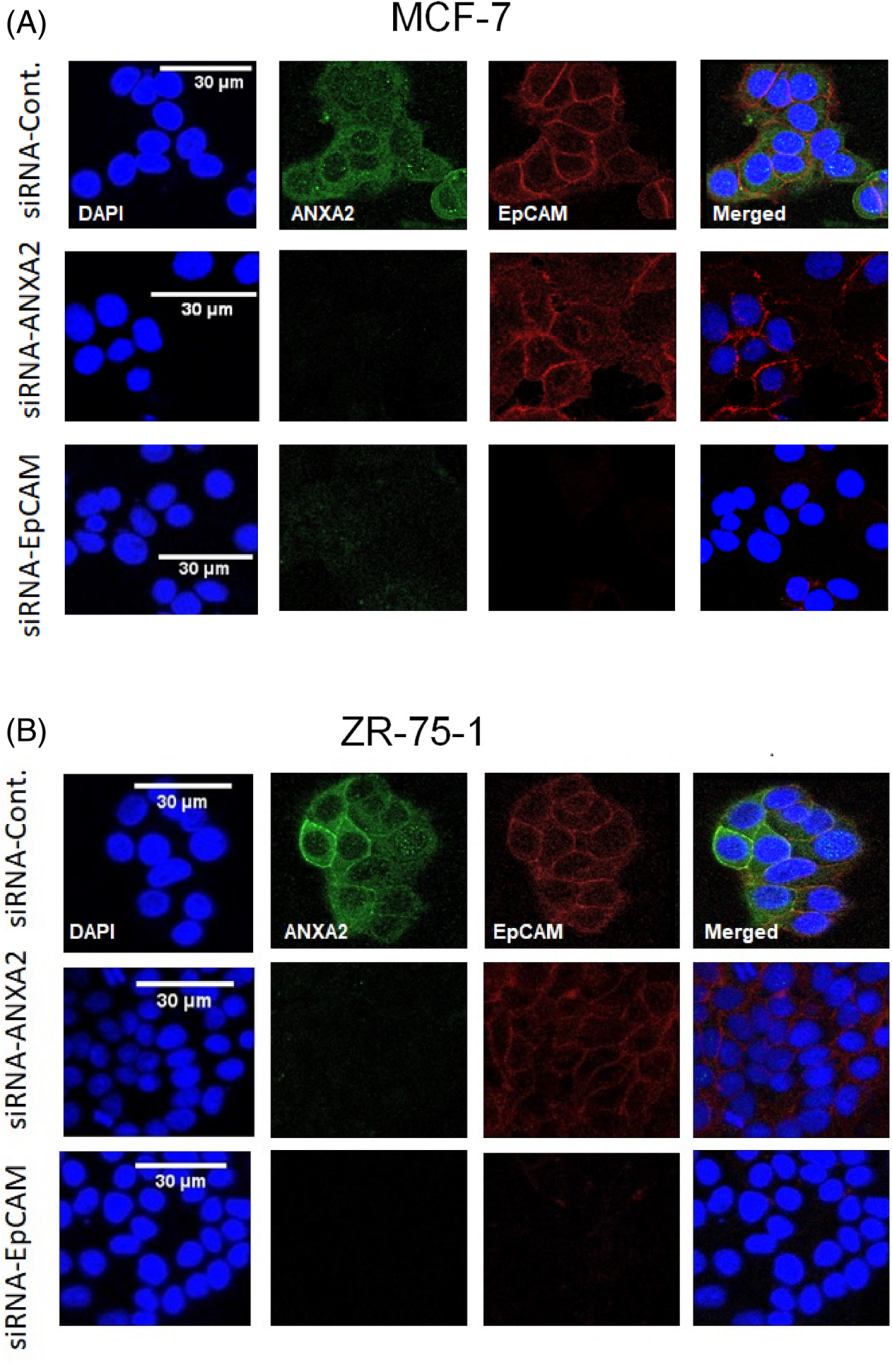
The images are representative of each respective group (*n* = 4), and the experiment was conducted twice and independently. (A) Effects of siRNA‐ANXA2 or siRNA‐EpCAM on ANXA2 and EpCAM by double immunofluorescence staining of MCF‐7: The signals of ANXA2 and EpCAM were lost upon siRNA‐ANXA2 and siRNA‐EpCAM treatments, respectively. In comparison to the control, the signal intensity of EpCAM was weaker due to siRNA‐ANXA2. However, due to siRNA‐EpCAM treatment, the localization and signal of ANXA2 were almost lost to a level comparable to when ANXA2 was targeted by siRNA‐ANXA2. (B) Effects of siRNA‐ANXA2 or siRNA‐EpCAM on ANXA2 and EpCAM by double immunofluorescence staining of ZR‐75‐1: The signals of ANXA2 and EpCAM were lost due to siRNA‐ANXA2 and siRNA‐EpCAM treatments, respectively. The signal intensity of EpCAM was weaker due to siRNA‐ANXA2, compared to the control. Due to siRNA‐EpCAM treatment, the localization and signal of ANXA2 were almost lost as if it was treated by siRNA‐ANXA2

Double immunofluorescence staining was performed again to study the subcellular localization of the two proteins after silencing ANXA2 or EpCAM. In MCF‐7 and ZR‐75‐1, siRNA‐ANXA2 did not affect the localization of EpCAM to the cell membranes despite that the signals of EpCAM were weaker mainly in ZR‐75‐1, compared to the controls (Figure 4(A),(B)). Upon siRNA‐EpCAM treatment of both MCF‐7 and ZR‐75‐1, however, the localization and signal of ANXA2 was almost lost to a level comparable to when ANXA2 was targeted by siRNA‐ANXA2 (Figure 4(A),(B)).

### 3.4 Effects of silencing EpCAM and/or ANXA2 on tPA

**FIGURE 5:**
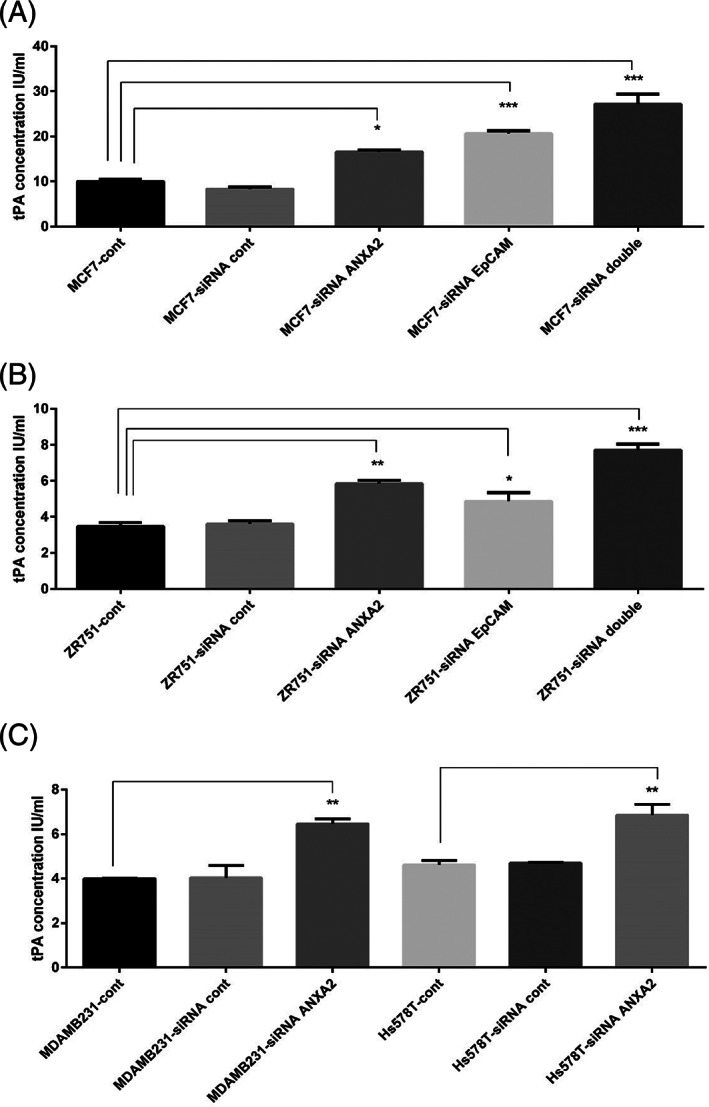
Each group was assessed in triplicates except control groups and standards were assessed in duplicates (*n* = 3). The values of *p* <.05 were considered significant whereas * indicates *p*‐value <.05, **p‐value <.01 and ***p‐value <.001. This experiment was performed twice and independently. (A) The levels of tPA concentration in MCF‐7: Silencing of ANXA2 or EpCAM led to a significant increase in the concentration of tPA in the cell culture supernatants, compared to the control. The concentration of tPA in the cell culture supernatants increased and magnified upon silencing both ANXA2 and EpCAM in the double siRNA group. (B) The levels of tPA concentration in ZR‐75‐1: siRNA‐ANXA2 or siRNA‐EpCAM resulted in a significant increase in the concentration of tPA in the cell culture supernatants, compared to the control. The concentration of tPA in the cell culture supernatants increased and magnified due to silencing both ANXA2 and EpCAM in the double siRNA group. (C) The levels of tPA concentration in MDA‐MB‐231 and Hs578T: In both cell types, the concentration of tPA in cell culture supernatants increased significantly due to siRNA‐ANXA2 treatment

In the cell culture supernatants of MCF‐7 and ZR‐75‐1, silencing of ANXA2 or EpCAM led to a significant increase in the concentration of unbound tPA in the cell culture supernatants, compared to the control (Figure 5(A),(B)). The concentration of unbound tPA in the cell culture supernatants increased and magnified upon silencing both ANXA2 and EpCAM as a double‐siRNA treatment (Figure 5(A),(B)). In the ERα^−/low^ and EpCAM^−/low^ cells, that are the MDA‐MB‐231 and Hs578T, the concentration of unbound tPA in the cell culture supernatants increased significantly due to siRNA‐ANXA2 treatment (Figure 5(C)).

## 4 DISCUSSION

The binding partners of EpCAM and the mechanisms by which EpCAM signals in and out of cells have been extensively studied but are not fully known. Therefore, the current study used EpCAM co‐immunoprecipitation followed by mass spectrometry and peptide fingerprinting to search for new potential binding partners of EpCAM. In the mass spectrometry analysis of ZR‐75‐1 EpCAM immunoprecipitates, a number of proteins were identified as co‐precipitated with EpCAM, and those proteins showing a statistically significant score in the identification process are listed in Table 3. It was appreciated that several of those proteins are abundant cellular proteins and can be viewed as background. However, the list included cytoskeletal proteins characteristic of cultured epithelial cells, and other proteins that are involved in several biological functions such as ANXA2. Additionally, one of the identified proteins was fatty acid synthase (FASN), which was previously reported to associate with EpCAM.^29^

For confirmation of protein co‐localization, we selected proteins that may contribute important novel functional mechanisms if a direct or indirect association with EpCAM can be confirmed. ERAP2 was identified by our group in a previous publication,^6^ and here we chose to study ANXA2 further. This is because the association between ANXA2 and EpCAM, and the biological/functional significance of this association are presently unknown.

EpCAM protein, as our group reported previously,^6^ is formed of 314 amino acids, and there are three domains: extracellular (the largest), transmembrane and cytoplasmic domain (Figure 1(A)). The ANXA2 protein is formed of 339 amino acids, and the amino acid sequence is shown in the Table 5. In the N–terminal domain, the domain for S100A10/p11 binding as well as known sites for regulatory tyrosine phosphorylation by pp60src and serine phosphorylation by PKC are all localized (Figure 1(B)). The assessment of the protein levels of ANXA2 and EpCAM relative to GAPDH showed that ANXA2 is expressed in all breast cancer cell lines whilst EpCAM is expressed only in the ERα^+^ cells ZR‐75‐1 and MCF‐7 (Figure 1(C)). The latter observation is consistent with what has been reported previously.^4^, ^30^

**TABLE 5:** Amino acid sequence of ANXA2 showing tyrosine and serine phosphorylation sites by pp60src and PKC, respectively

*Amino terminus*							
10	20	24	26	30	40	50	60
MSTVHEILCK	LSLEGDHSTP	PSAYGSVKAY		TNFDAERDAL	NIETAIKTKG	VDEVTIVNIL	
70	80	90		100	110	120	
TNRSNAQRQD	IAFAYQRRTK	KELASALKSA		LSGHLETVIL	GLLKTPAQYD	ASELKASMKG	
130	140	150		160	170	180	
LGTDEDSLIE	IICSRTNQEL	QEINRVYKEM		YKTDLEKDII	SDTSGDFRKL	MVALAKGRRA	
190	200	210		220	230	240	
EDGSVIDYEL	IDQDARDLYD	AGVKRKGTDV		PKWISIMTER	SVPHLQKVFD	RYKSYSPYDM	
250	260	270		280	290	300	
LESIRKEVKG	DLENAFLNLV	QCIQNKPLYF		ADRLYDSMKG	KGTRDKVLIR	IMVSRSEVDM	
310	320	330		339			
LKIRSEFKRK	YGKSLYYYIQ	QDTKGDYQKA		LLYLCGGDD			
*Carboxy terminus*							

*Note*: Amino terminus and S100‐10A binding site.

Abbreviations: S, Serine (AA 26) phosphorylation by PKC; Y, Tyrosine (AA 24) phosphorylation by pp60src.

Double immunofluorescence stains on EpCAM^+^ cells showed that the two proteins co‐localized mainly in a membranous pattern. Using siRNA‐ANXA2, the signal intensity of EpCAM was weaker. However, by using siRNA‐EpCAM treatment, the localization and signal of ANXA2 was almost lost to a level comparable to when ANXA2 was targeted by siRNA‐ANXA2 (Figure 4(A),(B)). Regarding the latter observation, it was thought that losing the localization and signal of ANXA2 due to siRNA‐EpCAM might be related to the secretion of ANXA2 to extracellular space or medium.^31^ However, the relative expression of ANXA2 at both mRNA (Figure 3(A)) and protein level (Figure 3(C),(D)) was significantly reduced due to siRNA‐EpCAM. In addition to that, the si‐RNA‐ANXA2 treatment led to a significant reduction at mRNA level of EpCAM (Figure 3(B)). Despite the protein levels of EpCAM were not affected significantly (Figure 3(D),(F)) by siRNA‐ANXA2, these results suggest that EpCAM has a regulatory function on the expression and localization of ANXA2 as well as that ANXA2 and EpCAM may be involved in regulating the expression of each other.

The current study also assessed the amount of unbound tPA in the supernatant of the cells after silencing ANXA2 and/or EpCAM. This is because ANXA2 has been reported to function as a co‐receptor for tissue plasminogen activator (tPA) at the cell surface of endothelial and cancer cells,^20^, ^32^, ^33^ and the current study suggests the co‐localization, dependence and interaction between ANXA2 and EpCAM at the cell surface. Our results showed that silencing of ANXA2 or EpCAM in the ERα^+^ cells led to a significant increase in the concentration of unbound tPA in the cell culture supernatants, compared to the control (Figure 5(A),(B)). Moreover, the concentration of unbound tPA in the cell culture supernatants increased and magnified upon silencing of both ANXA2 and EpCAM as a double‐siRNA treatment (Figure 5(A),(B)). These results indicated that ANXA2 may serve as a co‐receptor for tPA in an EpCAM‐dependent manner, since absence of the co‐receptor ANXA2 or its partner EpCAM led to accumulation of tPA in the supernatant as unbound tPA, which in turn may be associated with low plasmin generation.^33^ During assessment of the unbound tPA, the ERα^−/low^ and EpCAM^−/low^ cells, MDA‐MB‐231 and Hs578T, were included as a control. The results showed that the concentration of tPA in the cell culture supernatants of MDA‐MB‐231 and Hs578T increased significantly due to siRNA‐ANXA2 treatment (Figure 5(C)). This is consistent with a previous study, which has assessed tPA after silencing of ANXA2 in MDA‐MB‐231.^33^

It is known that ANXA2 has diverse functions, and it is involved in cancer cell motility, invasion, and metastases.^34^, ^35^, ^36^, ^37^, ^38^ Moreover, the up‐regulation of ANXA2 expression in cancer was directly correlated with advanced clinical stage^39^, ^40^; and higher ANXA2 expression was also observed in metastatic breast and colon cancer cells compared with the non‐metastatic cells.^36^, ^41^ All of these different roles of ANXA2 were linked in different reports to ANXA2‐tPA‐dependent plasmin generation.^20^, ^32^, ^33^, ^42^

In conclusion, the current study presents novel findings that may add insights for a better understanding of the complicated roles of EpCAM and ANXA2, which have been investigated and studied separately for decades. These findings include (1) ANXA2 is an interacting partner of EpCAM in the EpCAM^+^ ERα^+^ breast cancer cells; (2) ANXA2 co‐localizes with EpCAM at the plasma membrane of EpCAM^+^ ERα^+^ breast cancer cells; and (3) This co‐localization is of functional significance since EpCAM appeared to support ANXA2 to function as a co‐receptor for the tPA, and EpCAM seemed to have a regulatory influence on the expression/sub‐cellular localization of ANXA2.

## CONFLICT OF INTEREST

The authors declare no conflict of interest.

## AUTHOR CONTRIBUTIONS

All authors had full access to the data in the study and take responsibility for the integrity of the data and the accuracy of the data analysis. *Conceptualization*, C.E., M.N.; *Methodology*, S.M.A.‐Q., S.E.G., M.G., D.H.; *Investigation*, S.M.A.‐Q., S.E.G., M.G., C.E.; *Formal Analysis*, S.M.A.‐Q., S.E.G.; *Resources*, C.E., D.H., M.N.; *Writing—Original Draft*, S.M.A.‐Q., S.E.G., M.G., D.H.; *Writing—Review & Editing*, D.H., M.N.; *Supervision*, C.E., D.H., M.N.; *Funding Acquisition*, M.N.; *Data Curation*, S.M.A.‐Q., S.E.G.; *Validation*, S.M.A.‐Q., M.G.; *Project Administration*, D.H., M.N.

## ETHICAL STATEMENT

Not applicable.

## Supporting information


**Supplementary Figure S1** Scanned gel photo after EpCAM co‐immunoprecipitation *and before the mass spectrometry*. *The gel shows ANXA2 bands in MCF‐7*, *ZR‐75‐1* but not in MDA‐MB‐231 and Hs578T.

**Supplementary Figure S2** Each group was assessed in triplicates (mRNA levels) or duplicates (protein levels) (n = 4). The values of p <0.05 were considered significant whereas * indicates p‐value <0.05, ** p‐value <0.01 and ***p‐value <0.001. A) The mRNA levels of ANXA2 in MDA‐MB‐231 and Hs578T with and without siRNA‐ANXA2 treatment. B) Immunoblotting shows protein levels of ANXA2 and GAPDH in MDA‐MB‐231 and Hs578T with and without siRNA‐ANXA2 treatment. C) The protein levels of ANXA2 normalized to GAPDH in MDA‐MB‐231 and Hs578T with and without siRNA‐ANXA2 treatment.

## Data Availability

The data that support the findings of this study are available from the corresponding author upon reasonable request.
