# Supplementary figures and images for: The association between Annexin A2 and epithelial cell adhesion molecule in breast cancer cells
Source: Cancer Rep (Hoboken). 2021 Jul 9;5(5):e1498. doi: 10.1002/cnr2.1498 (PMC9124509; doi:10.1002/cnr2.1498)

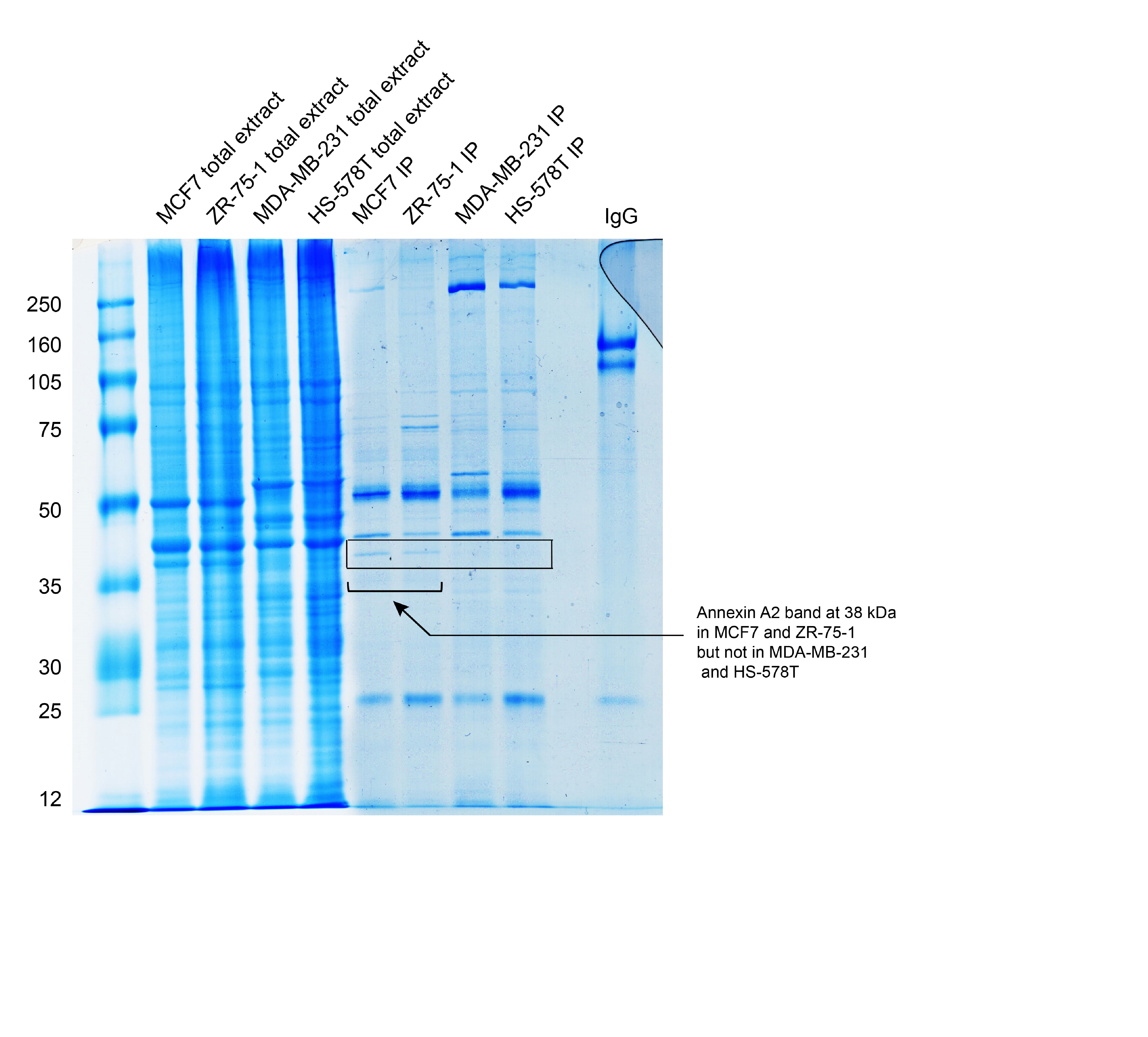

Supplement: Supplementary file 1 — Supplementary Figure S1 Scanned gel photo after EpCAM co‐immunoprecipitation and before the mass spectrometry. The gel shows ANXA2 bands in MCF‐7, ZR‐75‐1 but not in MDA‐MB‐231 and Hs578T. [file CNR2-5-e1498-s002.jpg]

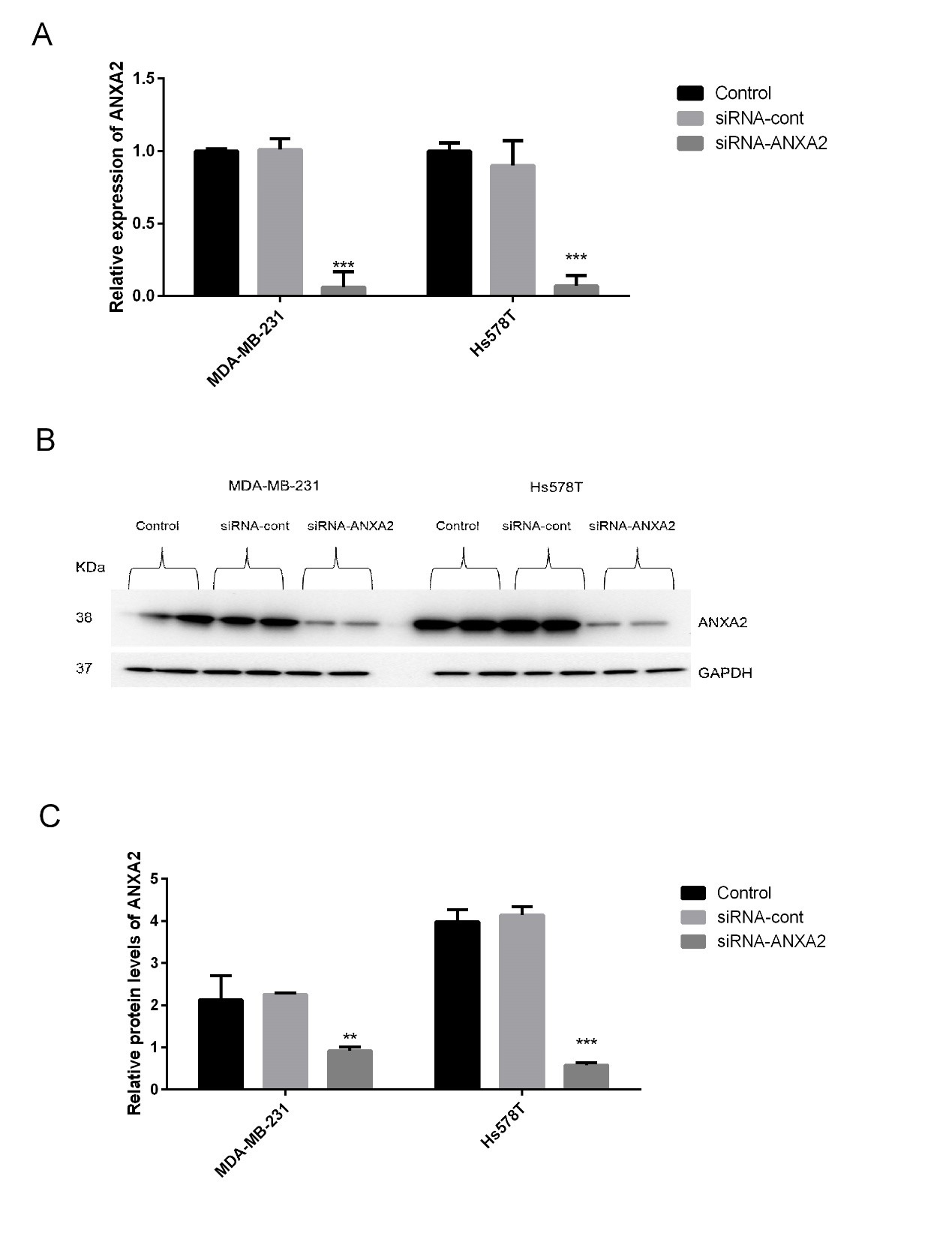

Supplement: Supplementary file 2 — Supplementary Figure S2 Each group was assessed in triplicates (mRNA levels) or duplicates (protein levels) (n = 4). The values of p <0.05 were considered significant whereas * indicates p‐value <0.05, ** p‐value <0.01 and ***p‐value <0.001. A) The mRNA levels of ANXA2 in MDA‐MB‐231 and Hs578T with and without siRNA‐ANXA2 treatment. B) Immunoblotting shows protein levels of ANXA2 and GAPDH in MDA‐MB‐231 and Hs578T with and without siRNA‐ANXA2 treatment. C) The protein levels of ANXA2 normalized to GAPDH in MDA‐MB‐231 and Hs578T with and without siRNA‐ANXA2 treatment. [file CNR2-5-e1498-s001.jpg]
